# Effect of Branched-Chain Amino Acid Supplementation Alone or Combined With Tryptophan or Methionine on Appetite Control and Related Health Outcomes in Older Adults: Protocol for a Randomized Controlled Trial

**DOI:** 10.2196/82436

**Published:** 2026-05-21

**Authors:** Shulin Zhang, Rosilene Ribeiro, David Raubenheimer, Corinne Caillaud, Jian Tan, Samantha Solon-Biet, Peter A Cistulli, Stephen J Simpson

**Affiliations:** 1 School of Life and Environmental Science, Faculty of Science, The University of Sydney, Johns Hopkins Dr, Camperdown, 2050, Australia, +61 (02) 93519646; 2 Charles Perkins Centre, The University of Sydney, Sydney, Australia; 3 School of Medical Sciences, Faculty of Medicine and Health, The University of Sydney, Sydney, Australia; 4 Sydney Medical School Nepean, The University of Sydney, Sydney, Australia; 5 Nepean Blue Mountains Local Health District, Nepean Hospital, Sydney, Australia; 6 Faculty of Medicine and Health, Northern Clinical School, University of Sydney, Sydney, Australia; 7 Department of Respiratory and Sleep Medicine, Royal North Shore Hospital, Sydney, Australia

**Keywords:** branched-chain amino acids, aging, obesity, appetite, dietary intervention

## Abstract

**Background:**

Branched-chain amino acids (BCAAs) are essential amino acids for protein metabolism. Preclinical research in mice suggested that BCAA intake relative to other amino acids, in the context of a high-carbohydrate diet, was associated with hyperphagia, obesity, and reduced lifespan. These effects were not attributed to BCAAs alone, nor did they manifest through canonical mechanistic target of rapamycin–insulin-like growth factor 1 pathways; rather, they resulted from indirect effects of other amino acids, notably tryptophan, on appetite. As population aging and obesity-related chronic diseases present significant public health challenges, understanding appetite regulation is critical. To date, no clinical trial has examined the effects of BCAAs on appetite regulation in older adults. On the basis of our preclinical results, we hypothesized that, compared to the control diet, a diet supplemented either with BCAA or with BCAAs and methionine would increase appetite and energy intake, whereas supplementation with BCAA and tryptophan would not increase appetite.

**Objective:**

We aimed to translate these preclinical findings to humans by examining the effects of BCAAs per se and in combination with tryptophan and methionine on appetite and other health measures in a cohort of older participants.

**Methods:**

This randomized controlled clinical trial recruited 110 adults (aged 65-80 y; BMI 20-35 kg/m^2^). Participants were randomly allocated to four 4-week intervention groups: (1) control (no supplementation), (2) BCAAs, (3) BCAAs+tryptophan, or (4) BCAAs+methionine. All participants received a controlled diet, with intervention groups additionally receiving amino acid supplements. The primary outcomes are appetite assessed via self-reports and fibroblast growth factor 21 levels (a marker of protein appetite), and energy intake quantified from dietary intake data. Secondary outcomes include body composition, cardiometabolic health, gut microbiota, blood biomarkers, sleep, and physical performance. Descriptive statistics will be used to summarize participant characteristics. Linear mixed models will assess intervention effects, with and without adjustment for relevant covariates. Diet-specific self-reported appetite and palatability scores will be analyzed using generalized additive mixed models.

**Results:**

The trial was registered on April 12, 2021. Recruitment commenced in April 2022 and was completed in November 2025, with 308 individuals screened and 100 completing the study. Data analyses are planned for completion by December 2026, with results expected to be published in 2027. Data cleaning and analysis are currently in progress and are expected to be completed by December 2026, with trial results expected to be published in 2027.

**Conclusions:**

This study will clarify the effects of BCAAs, either alone or in combination with tryptophan or methionine, on appetite and related outcomes in an older population. The findings may inform nutritional strategies targeting appetite regulation and metabolic health to support healthy aging.

## Introduction

Population aging represents one of the major global public health challenges. It has been estimated that by 2030, 1 in 6 people in the world will be aged ≥60 years [1]. With population aging, the prevalence of obesity-related chronic diseases is also rising, including type 2 diabetes, cardiometabolic diseases, obesity, and sarcopenia [2]. These conditions significantly impact quality of life and health care costs. Dietary interventions involving supplementary food products have been prominently highlighted in research over the past decades and, in some cases, have demonstrated positive effects in supporting metabolic health and healthy aging [3-5].

Protein is composed of amino acids (AAs) that impact all aspects of metabolism, growth, development, and aging [6]. Accordingly, many organisms regulate the intake and metabolism of protein, with a specific appetite for protein representing an important determinant of feeding behavior. It has been shown that many species, including humans, consume more energy when dietary protein is diluted by fats and carbohydrates, a phenomenon known as protein leverage, thereby increasing the risk of weight gain, obesity, and adverse health outcomes [7,8]. Although a low concentration of dietary protein increases the risk of deleterious health consequences through increased food and energy intake, excess protein intake has also been shown to have detrimental effects. High dietary protein, especially when associated with low intake of healthy carbohydrates, increases markers of aging in model organisms, such as mechanistic target of rapamycin (mTOR) and insulin-like growth factor 1 (IGF-1), especially during midlife [9,10]. Additionally, it has been demonstrated that changes in the dietary ratio of protein to carbohydrate impact several health markers including reproduction, immune function, microbiome, and late-life cardiometabolic health [9,11].

Essential AAs are 9 of the 20 AAs that must be obtained through the diet [12]. The branched-chain AAs (BCAAs; ie, valine, leucine, and isoleucine) are well known for their established anabolic and anticatabolic effects on skeletal muscle protein metabolism. These properties have made BCAA supplementation attractive to athletes and older adults, who commonly experience anabolic resistance and age-related loss of lean body mass. By contrast, circulating levels of BCAAs have also been considered predictors of developing type 2 diabetes, insulin resistance, and obesity [13,14]. Although elevated BCAA levels are associated with increases in canonical markers of aging, such as mTOR and IGF-1, this occurs primarily when BCAAs are provided against a low-carbohydrate background [9].

In mice, it was found that when BCAAs were increased in the diet within a fixed protein complement against a high-carbohydrate background, this resulted in hyperphagia, obesity, and reduced lifespan [15]. However, these effects were not associated with BCAA-activating proaging nutrient-sensing pathways such as mTOR, but rather were due to indirect effects on appetite control mediated by other AAs within the protein complement. It was found that selectively increasing dietary tryptophan and threonine, but not methionine, prevented hyperphagia under high-BCAA intake. Increasing the proportion of BCAAs within the fixed complement of dietary protein inevitably increased the ratio of BCAAs with respect to other AAs. Because BCAAs and tryptophan share an AA transporter (L-type AA transporter 1) for entry into the brain, competition from elevated BCAA with tryptophan led to a reduction in brain tryptophan concentrations, reduced serotonin levels, and consequently increased appetite signaling [15-17].

Fibroblast growth factor 21 (FGF-21) is a peptide hormone involved in metabolic homeostasis and is predominantly produced by the liver [18]. FGF-21 is the first known hormone signal for low protein status, being elevated in circulation under low-protein conditions. A high-carbohydrate, low-protein diet has been shown to boost levels of FGF-21. Perfusion of FGF-21, either centrally or peripherally, results in mice selecting a higher protein intake, indicating its primary role in the control of protein appetite [19].

To date, human studies of BCAA supplementation have primarily focused on metabolic health, muscle protein synthesis, liver disease, and neurological or sleep-related outcomes, demonstrating the feasibility and safety of BCAA interventions [20-24], while the effect of BCAA, alone or in combination with other AAs, on appetite regulation in older adults remains largely unexplored. This gap is clinically important: aging is associated with the “anorexia of aging” (contributing to malnutrition) as well as dysregulated appetite responses that trigger conditions such as sarcopenic obesity [25,26].

Our aim in this trial is to translate the preclinical work from aging mice to humans aged 65 to 80 years by supplementing a standard control diet (18%-19% of energy from protein, 30%-35% from fat, and 45%-65% from carbohydrates) with BCAA alone or in combination with either tryptophan or methionine. We hypothesize that, compared to the control diet, participants receiving a diet supplemented with BCAAs alone or BCAAs in combination with methionine will have increased appetite and energy intake, whereas supplementation with BCAAs and tryptophan will mitigate these effects.

## Methods

### Study Design

**Figure 1.**
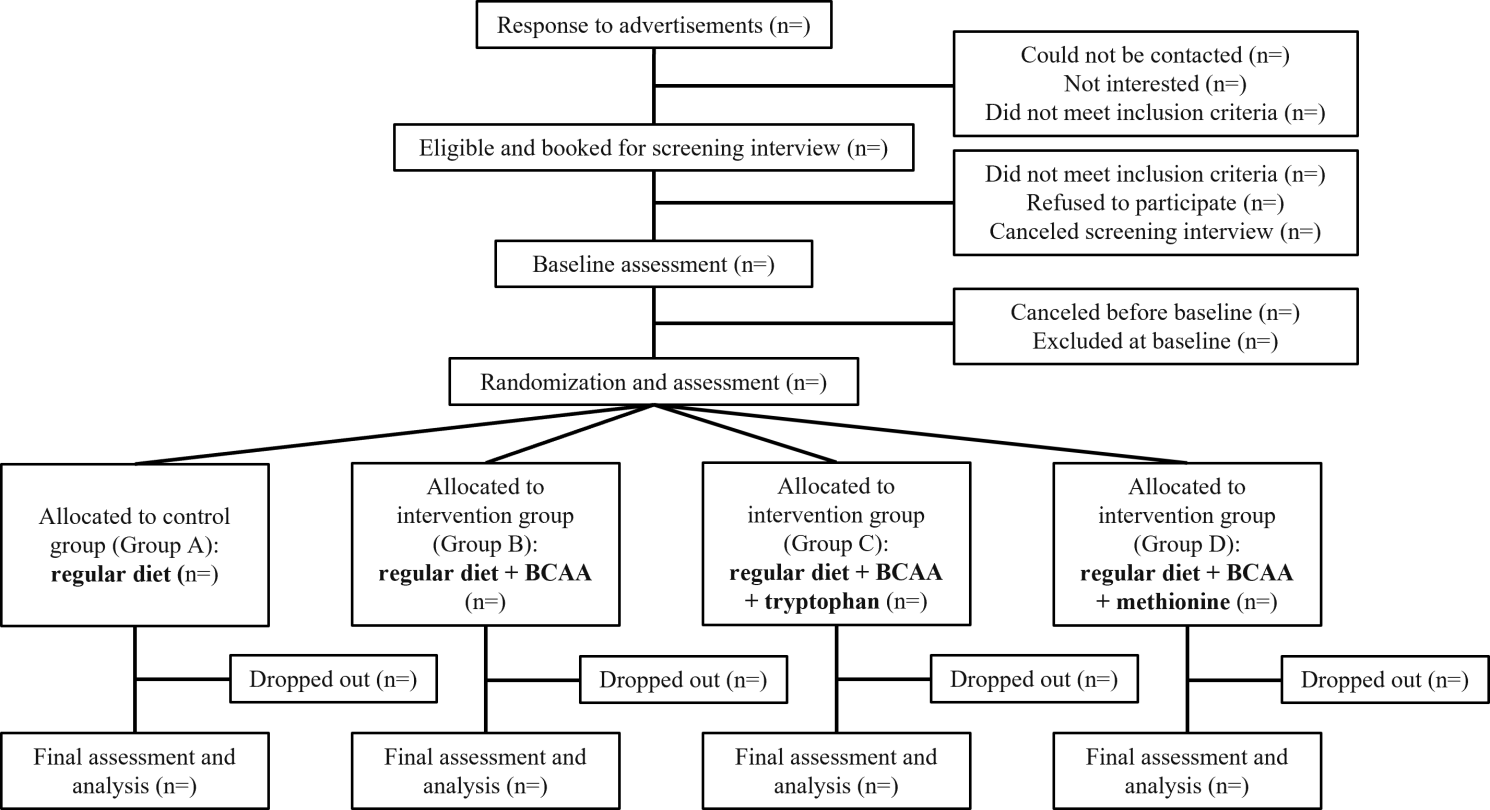
CONSORT (Consolidated Standards of Reporting Trials) flow diagram of participant recruitment, randomization, and analysis. BCAA: branched-chain amino acid.

The Optimizing Diet for Better Health (OptHealth) study is a randomized clinical trial (RCT) with a total target sample of 110 participants and 100 completers. The sample size was determined by circulating FGF-21 levels (pg/mL), using a 2-tailed ANOVA design and assuming an SD of 70 [18], 80% power, and an α of .05; a difference of 30% (control group mean 149.4 pg/mL) [27] may be detected with a sample size of 25 participants per group (100 in total). The dropout rate was estimated to be 10%; therefore, 110 participants were recruited. Clinical assessments were performed prior to initiating the intervention and after the 4-week intervention period (Figure 1). All study visits were conducted at the Charles Perkins Centre (CPC)–Royal Prince Alfred Hospital (RPAH) clinic. This study protocol was prepared in accordance with the SPIRIT (Standard Protocol Items: Recommendations for Interventional Trials) guidelines for RCTs [28] (Checklist 1).

### Eligibility Criteria

Trial inclusion and exclusion criteria are presented in Textbox 1.

The exclusion criteria were selected to minimize confounding effects on appetite regulation, energy intake, and metabolic outcomes and also to ensure participant safety and protocol compliance. Individuals with metabolic, endocrine, hepatic, renal, or malignant conditions were excluded due to their potential influence on appetite, protein metabolism, and cardiometabolic biomarkers. Individuals with known disorders of BCAA metabolism, such as maple syrup urine disease, were excluded due to their altered BCAA metabolism and potential safety concerns associated with BCAA supplementation [29]. Participants using medications known to affect body weight, appetite, or energy expenditure were excluded to reduce pharmacological confounding. Specifically, individuals who had used antibiotics within 4 weeks prior to study commencement were excluded due to their potential impact on gut microbiota and related outcomes. Individuals with recent unintentional weight loss, current smoking, or high alcohol intake were excluded due to their independent effects on appetite regulation and metabolic health. Participants following restrictive dietary patterns or with food allergies or intolerances that would limit adherence to the provided study diet were excluded to ensure dietary intervention standardization.

Textbox 1. **Inclusion and exclusion criteria for the Optimizing Diet for Better Health study.**

**Inclusion criteria**

- Male participants and female participants (at postmenopausal stage) aged 65 to 80 years
- BMI of 20 to 35 kg/m^2^
- Access to and ability to operate a smartphone (running iOS or Android)
- Ability to provide informed consent and to undergo all study assessments

**Exclusion criteria**

- Diagnosis of diabetes mellitus (insulin-dependent; type 1 or type 2), renal or liver disease, cancer or active neoplasms, untreated or uncontrolled hyperthyroidism, schizophrenia, or sleep apnea or known disorders of branched-chain amino acid metabolism (maple syrup urine disease)
- Use of antibiotics or other medications known to affect weight or energy expenditure within the 4 weeks prior to baseline assessment
- Unintentional weight loss of >10% body weight within the past 5 years
- Current smoking
- Alcohol intake of >3 standard drinks per day or unwillingness to refrain from alcohol
- Adherence to a vegan and vegetarian diet, strict dietary restrictions, food allergies and/or intolerances, and contraindications to dietary changes as determined by a treating physician

### Recruitment

Participants were recruited via flyers, the University of Sydney website, community and library engagement events, senior fitness classes, and word of mouth. Interested individuals contacted the research team via phone call or email. A prescreening assessment was conducted over the phone to determine their initial eligibility. Those who meet the criteria were invited to attend a face-to-face screening interview at the CPC-RPAH clinic.

### Screening and Consent

During the screening visit (visit 1), potential participants were asked questions to gather information on sociodemographic characteristics, medical history, medication use, smoking, and alcohol consumption. The screening interview was conducted by a study investigator (research dietitian), who provided all the information related to baseline and final assessments, including information about blood, stool, and urine sample collection; weighed food diary completion; and use of a wrist-worn device to measure physical activity level and sleep quality and duration. Written informed consent was obtained from participants who chose to enroll in the study.

### Baseline and Final Assessment

**Figure 2.**
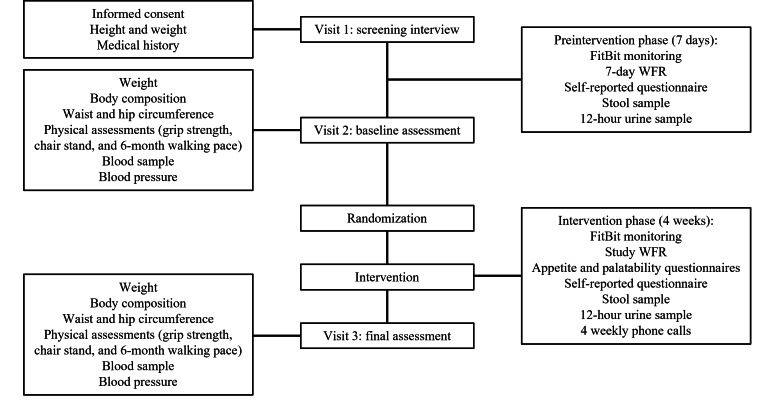
Schedule of visits and testing procedures at the Charles Perkins Centre Royal Prince Alfred Hospital clinic of Optimizing Diet for Better Health study. WFR: weighted food record.

Following the screening interview, participants attended another 2 clinical assessment visits (visits 2 and 3), during which several health-related measurements were taken. Details on the schedule of visits and health assessments are outlined in Figure 2.

### Diet

During the 4-week intervention period, participants were provided with ad libitum access to a standard diet containing 18% to 19% of energy from protein, 30% to 35% from fat, and 45% to 65% from carbohydrates, as per the Acceptable Macronutrient Distribution Ranges set by the National Health and Medical Research Council [30]. Participants were allowed to consume additional nonstudy foods, including milk (up to 100 ml a day), nonstarchy vegetables (up to 2 cups a day), and fresh fruit (up to 2 servings a day). A bottle of 1 L of olive oil was given to participants for salad dressing.

The number of serves of core food groups within the study diet is displayed in Table 1. The prescribed study diet provided approximately 6700 kJ per day, supplying 85 g of protein, 45 g of fat, and 200 g of carbohydrates (detailed nutrition profile is provided in Multimedia Appendix 1). When including the permitted nonstudy foods, the total daily food provision was estimated to provide approximately 8200 to 8700 kJ per day, with corresponding macronutrient availability of approximately 90 to 93 g of protein, 60 to 75 g of fat, and 240 to 250 g of carbohydrates. All calculations were performed using FoodWorks 10 Professional for Windows (Xyris Software Pty Ltd) [31].

**Table 1.** Servings of food groups provided in the study diet.

Food group	Study diet (serves)
Grains	6.2
Whole grains	2.5
Other grains	3.7
Fruit	1.1
Vegetables	6.6
Legumes	0.1
Other vegetables	6.5
Diary	1.6
Milk	0.7
Cheese	0.3
Yoghurt	0.6
Protein foods	1.6
Red meats	0.9
Poultry	0.5
Eggs	0
Seafood	0.1
Nuts or seeds	0.1
Legumes protein	0
Oil equivalents (tsp)^a^	4

a LC n-3, long chain omega-3 fatty acids; tsp: teaspoon; oil equivalent: the amount of dietary fat or oil represented in the Australian Dietary Guidelines. One oil equivalent is typically equal to 10 grams of fat.

### Randomization and Intervention

Enrolled participants were randomized to 1 of 4 treatment groups using permuted block randomization. Random blocks of 4 were generated using the R package “blockrand” [32] for male and female participants separately. Allocation was concealed using sealed, opaque, numbered envelopes. The study coordinators and participants were not blinded to group allocation.

Participants randomized to intervention groups were provided with supplements to be taken with their meals (Table 2). Participants in the BCAA group (group B) were instructed to consume 1 capsule of BCAA supplements thrice daily with their main meal. Participants in the BCAA and tryptophan group (group C) were instructed to consume 1 capsule of BCAA thrice daily and 1 tablet of tryptophan supplement twice daily. Participants in the BCAA and methionine group (group D) were instructed to consume 1 capsule of BCAA and 1 capsule of methionine supplement thrice daily.

**Table 2.** Description of diets and interventions for study groups.

Diet name or group	Diet or intervention description
Group A: control	Regular diet containing 18% to 19% of total energy from protein
Group B: BCAA^a^	Regular diet+3 doses of BCAAs (L-leucine 300 mg, L-isoleucine 150 mg, and L-valine 150 mg)
Group C: BCAA+tryptophan	Regular diet+3 doses of BCAAs (L-leucine 300 mg, L-isoleucine 150 mg, and L-valine 150 mg)+2 doses of L-tryptophan (1000 mg)
Group D: BCAA+methionine	Regular diet+3 doses of BCAAs (L-leucine 300 mg, L-isoleucine 150 mg, and L-valine 150 mg)+3 doses of L-methionine (500 mg)

a BCAA: branched-chain amino acid.

### Primary Outcome Measurements

Appetite and total food intake were the primary outcomes in this study. They were measured using plasma FGF-21, ghrelin, self-reported appetite, and dietary intake.

#### Dietary Intakes

Participants’ dietary intake were recorded in 2 phases: a 7-day habitual intake period prior to the intervention and a 4-week intervention phase during which study-provided foods were consumed. To assess habitual dietary intakes, participants completed a 7-day weighed food record (7dWFR) prior to the dietary intervention. A kitchen scale (IKEA ORDNING) was supplied to each participant to complete the 7dWFR. Dietary information was recorded by participants manually in a 7-day food diary or via a mobile app (Research Food Diary) [33]. The 7dWFR was adapted from Henderson et al [34].

During the intervention phase, participants used a study dietary record booklet, which included a list of all study meals, to document their intake. Participants allocated to intervention groups also recorded supplement consumption throughout the intervention period in the same booklet. Individual dietary intakes (both habitual and intervention) were inputted into FoodWorks 10 Professional with the Australian Food, Supplement and Nutrient Database 2013 [31,35] and converted into nutrients intakes.

#### Blood Sample and FGF-21

Approximately 50 mL of fasting blood (20 mL serum and 30 mL plasma) was collected by a qualified blood collector at baseline and final assessment. Serum was collected into 8.5 mL serum separator tube II Advance tube (BD Vacutainer ref 367,958; Becton Dickinson). Cardiometabolic health was measured using serum markers, including electrolytes, urea, and creatinine; calcium and phosphate; protein and albumin; AAs; triglycerides, total cholesterol, and high-density lipoprotein cholesterol; glucose and insulin; C-reactive protein; IGF-1; leptin; and liver function markers, including alanine aminotransferase, aspartate aminotransferase, and lactate dehydrogenase.

Plasma was collected into 10 mL ethylenediaminetetraacetic acid tubes (BD Vacutainer; Becton Dickinson) for measurement of circulating proteins, including FGF-21, ghrelin, cytokines, and other relevant biomarkers. Targeted protein concentrations were quantified using enzyme-linked immunosorbent assay. EDTA blood sample will also be used for cytokine analysis. Additional blood samples will be collected for isolation of peripheral blood mononuclear cells for immune profiling.

#### Palatability and Appetite Questionnaires

Visual analog scale (10-cm horizontal line) questionnaires were used to determine participants’ palatability of study foods and appetite during the intervention [36]. Participants completed palatability and appetite questionnaires on every fourth day of each intervention week (days 4, 11, 18, and 25).

### Secondary Outcome Measurements

#### Self-Reported Questionnaire

Participants completed a set of questionnaires before and after the dietary intervention. These include a physical activity questionnaire, from which the Physical Activity Scale for the Elderly score was calculated, and the Sleep Apnea Global Interdisciplinary Consortium questionnaire, which assessed sleep quality [37,38]. Participants also self-reported their demographic details, medical history, tobacco and alcohol use, and daily habits through questionnaires before starting the study.

#### Physical Activity and Sleep Patterns

Participants were provided with Fitbit devices (version Fitbit Inspire 2; Google) to enable biometric data collection (daily step count, heart rate, and sleep duration and quality) through Fitabase’s proprietary API tool [39,40]. Participants reviewed Fitbit’s privacy policy during screening interview and agreed to Fitbit’s terms and conditions before making a study account. Device charging and data syncing were tracked through the Fitabase platform. Participants were instructed to wear the Fitbit watch 24 hours a day during both the baseline week (7-day preintervention phase) and the 4-week intervention period.

#### Blood Pressure

Systolic and diastolic blood pressure were measured twice, to the nearest 2 mm Hg, 1 min apart after 5 minutes of quiet rest in a seated position [41], using automated sphygmomanometers on the right arm of participants during baseline and final assessments.

#### Body Composition

Participants fasted overnight prior to body composition assessment. Body weight, fat mass, and fat-free mass were measured using air displacement plethysmography (BodPod; COSMED). To minimize potential error from isothermal air trapped in clothing and hair, participants wore tight-fitting clothes and a Lycra swim cap. Body fat percentage was determined by the Siri equation [42]. The procedure took no longer than 10 minutes.

#### Physical Capacity Assessment

Handgrip strength, repeated chair stand, and habitual walking speed were tested to assess participants’ physical performance at baseline and final assessments. Hand grip strength was assessed with participants seated on a straight-backed chair. The shoulders were positioned in a neutral abduction with the arms in a neutral rotational position. The wrists were positioned at 0° to 30° extension and 0° to 15° flexion. Participants were instructed to squeeze the dynamometer with maximum effort for a duration of 10 to 30 seconds. Assessments were conducted with both hands (2 measurements each) using the Jamar dynamometer (Promedics), with 30-second rest intervals. A chair without armrests and a seat height of 40 cm was used for the repeated chair stand assessment. The test involved measuring the time taken for participants to successfully complete 5 repeated chair stands without using their arms. Participants’ mobility was evaluated using a 6-meter walking test, during which each participant was asked to walk at normal speed over a 6-meter course [43].

#### Gut Microbiome and Short-Chain Fatty Acid

Stool samples were collected within 48 hours prior to baseline and final assessments. Participants were provided with stool sample collection kits, including a fecal container (Sarstedt feces tube, with blade, screw cap; 54×28 mm and white), a thermal bag (Thermabag), and a small ice pack during the screening interview and baseline assessment. Written instructions on sample collection, storage, and transportation were provided to participants during the screening interview. Participants recorded the time and date of the collection and stored the samples in the freezer immediately after collection. Participants were also asked to minimize the time samples remain out of the freezer to <2 hours when delivering them to the clinic on assessment day. Samples were stored at –80 °C immediately upon receipt.

Microbial DNA will be extracted using the SPINeasy DNA Pro Kit for Feces (MP Biomedicals), and bacterial community composition was analyzed via 16S ribosomal RNA gene sequencing targeting the V3-V4 region, following established methods previously described by Ribeiro et al [11]. Raw sequencing data will be processed using validated bioinformatics pipelines (DADA2) to generate amplicon sequence variants. Downstream analyses will be performed using R software (version 4.6.0; R Foundation for Statistical Computing) to assess microbial diversity and relative taxonomic abundance. Differences in overall gut microbiome composition and differences in specific taxa between intervention groups and across time points will be assessed using appropriate univariate, multivariate, and mixed-effects statistical approaches.

Fecal short-chain fatty acid (acetate, butyrate, and propionate) will be quantified using nuclear magnetic resonance, as previously described [11]. Short-chain fatty acid concentrations will be expressed relative to sample weight and analyzed to assess changes over time and between intervention groups.

#### Urinary Urea

Participants were provided with a 4-L urine container to collect a 12-hour urine sample prior to baseline and final assessments. Samples are stored at 4 °C environments following each collection. Total weight and volume are measured by the study investigator prior to sample aliquoting. Aliquoted samples are stored in an −80 °C fridge for further analysis. These samples will be used to assess participants’ protein intake. Urea concentrations will be quantified using standard enzymatic assays in an accredited laboratory, and total urinary urea excretion will be calculated based on urine volume and concentration.

#### Other Measures

Participants received weekly phone calls from a study investigator on every third day of the week during the intervention period. The purposes of the phone calls were to (1) remind participants to complete the palatability and appetite questionnaires on the following days, (2) ask participants how they managed their diet, and (3) ask if additional nonstudy foods were consumed.

### Monitoring

#### Data Management and Biospecimen Storage

A Research Data Management Plan has been created as per Sydney Local Health District guidelines. A 3-digit, deidentified number has been created and assigned to participants. All collected participant data are repersonalized using the assigned 3-digit number for the purposes of data storage. Deidentified data are stored securely on REDCap (Research Electronic Data Capture; Vanderbilt University), and backups are stored in a password-protected computer file within a secured area of the CPC and only accessed by study investigators. Hard copy files are stored in a securely locked area at CPC and accessed only by the study investigators.

Collected biospecimens were numbered sequentially. A sample manifest is maintained to link sample numbers to participant codes. All biospecimens are stored in secured temperature-monitored −80 °C freezers at CPC. The manifest includes the number of samples, date of collection, sample type, and amount. Sample analysis performed in an accredited laboratory may reveal information that may be important for the health of the participant. Health risk factors for metabolic diseases may be identified (glucose, insulin, triglycerides, total cholesterol, and high-density lipoprotein cholesterol), and participants will be informed if abnormal results are identified.

#### Adverse Events

This study has been identified as a relatively low-risk study. Participants were instructed to contact emergency services immediately if they experienced any food allergy or intolerance symptoms during the study and to contact the study coordinator as soon as possible to notify them of the incident. Any unexpected and related serious adverse events arising from this study were reported to the Human Research Ethics Committee in accordance with relevant guidelines.

### Statistical Analysis

All statistical analyses will be conducted using R software, with significance set at *P*<.05. The analysis will follow the following two approaches: (1) within-subject comparisons using paired sample *t* tests (via the “t.test” function in stats package) to assess overall changes from baseline (habitual diet) to after intervention and (2) between-group analyses of RCT findings across all 4 groups, maintaining treatment assignment integrity and excluding dropouts.

A 1-way ANOVA will be used to examine across the 4 diet groups in follow-up measurements. A linear model (model 1) will be fitted with follow-up values as the outcome and baseline measurements as covariates to assess the effect of the intervention. A second model (model 2) will further adjust for potential confounders, including age, sex, BMI, and physical activity level.

Self-reported appetite and palatability data specific to each diet will be analyzed using the R package *mgcv* with the gam function [44]. Generalized additive mixed models will be applied to assess the effects of dietary interventions on appetite scores. For BCAA levels and metabolites, relative changes (fold changes) will be calculated as the ratio of final to baseline values. A 1-sample *t* test (null hypothesis of fold change=1) will be used to test whether the change from baseline is significant compared to final. To compare differences in fold change across groups, models will be fitted using follow-up measurements as outcomes, adjusting first for baseline values. A second model will further adjust for covariates such as age, BMI, sex, and physical activity levels. Dropouts will be treated by exclusion for analysis.

### Ethical Considerations

The study was conducted in accordance with the National Statement on Ethical Conduct in Human Research (2007; updated 2018) [45], the CPMP-ICH Note for Guidance on Good Clinical Practice [46], and the principles of the Declaration of Helsinki. Compliance with these standards provides assurance that the rights, safety, and well-being of trial participants are respected. The study was approved by Ethics Review Committee (RPAH Zone) of the Sydney Local Health District (X20-0463 – 2020/STE04496). The trial was registered with the Australian New Zealand Clinical Trials Registry (ACTRN12621000402842). Written informed consent was obtained from all participants prior to enrollment and before any study-related procedures were conducted.

Participant data are collected and stored in a deidentified format using a unique, randomly generated study identification code assigned to each participant. Identifiable information is stored separately from study data and is accessible only to authorized study investigators. All electronic data are stored on secure, password-protected systems in accordance with institutional data governance policies. There are no costs associated with participation in the study, and participants do not receive financial compensation.

## Results

Recruitment commenced in April 2022, with the original recruitment period planned for completion within 2 years. However, recruitment was delayed due to COVID-19–related lockdowns and associated restrictions. Enrollment was completed in November 2025.

In total, 208 individuals were screened, of whom 195 were eligible to attend the screening interview. Following screening, 109 participants were enrolled and randomized to treatment groups. Of these, 100 participants completed the 4-week intervention, and 9 were lost to follow-up.

Participants’ baseline characteristics are summarized in Table 3; no statistically significant differences were observed across the 4 intervention groups.

**Table 3.** Participants’ baseline characteristics by randomly assigned diet treatment group.

Characteristic	Overall (n=100)	Control (n=25)	BCAA^a^ (n=24)	BCAA+tryptophan (n=26)	BCAA+methionine (n=25)
Age (years), mean (SD)	71.1 (4.2)	71.2 (4.5)	70.7 (4.1)	71.8 (4.3)	70.6 (4.1)
BMI (kg/m^2^), mean (SD)	27.3 (4.0)	27.8 (3.3)	27.8 (4.6)	27.5 (4.3)	26.2 (3.7)
Physical Activity Scale for the Elderly (points), mean (SD)	146.3 (67.9)	157.2 (66.2)	157.2 (65.8)	116.8 (68.6)	155.5 (65.9)
Weight (kg), mean (SD)	74.4 (12.3)	75.1 (9.8)	76.8 (11.6)	73.1 (13.9)	72.8 (13.7)
Body fat (%), mean (SD)	38.9 (8.9)	40.3 (8.6)	38.3 (11.4)	39.4 (7.1)	37.4 (8.2)
Fat mass (kg), mean (SD)	29.1 (9.0)	30.2 (7.5)	29.8 (11.3)	29.1 (8.7)	27.3 (8.4)
Grip strength (kg), mean (SD)	26.6 (9.0)	26.1 (9.8)	27.5 (10.0)	26.9 (8.2)	26.0 (8.4)
High education^b^, n (%)	90 (90.0)	22 (88.0)	22 (91.7)	23 (88.5)	23 (92.0)
Ex-smoker, n (%)	41 (41.0)	9 (36.0)	8 (33.3)	11 (42.3)	13 (52.0)
Born in Australia, n (%)	66 (66.0)	17 (68.0)	14 (58.3)	19 (73.1)	16 (64.0)

a BCAA: branched-chain amino acid.

b High education includes any education obtained after high school, whereas low education includes completion of high school or below.

All outcome data collection has been completed. Data cleaning and statistical analyses are scheduled to commence in March 2026, with final analyses expected to be completed by December 2026. Results from this trial are anticipated for publication in 2027.

## Discussion

### Principal Findings

In this paper, we have outlined the protocol for the study design, recruitment, collection, and processing of health assessment data related to the OptHealth study. As the global population ages, dietary interventions are increasingly used as a critical tool for managing metabolic health and age-related diseases. Previous research using mouse models demonstrated that a diet with high concentrations of BCAAs resulted in hyperphagia and associated obesity. Notably, increased BCAAs combined with tryptophan suppressed food intake, whereas BCAAs and methionine did not. The OptHealth study is designed to translate the preclinical work to assess the short-term effects of BCAA supplementation alone or in combination with tryptophan or methionine on appetite and other health outcomes in a healthy population of older adults. We expect the findings from this study to enhance our knowledge of dietary AA profile and appetite regulation, potentially contributing to the development of practical dietary strategies to support health span.

### Comparison to Prior Work

Existing BCAA-related clinical trials in older adults have primarily focused on muscle protein synthesis, insulin sensitivity, and fat metabolism—central factors in addressing conditions such as sarcopenia, type 2 diabetes, and obesity. However, despite the growing body of research, the relationships between BCAAs and appetite regulation remain unclear, particularly given the significant physiological overlap between appetite control and the development of chronic diseases [47,48].

Additionally, previous human trials investigating BCAA supplementation have commonly administered higher absolute doses, particularly in the context of exercise performance and muscle protein synthesis, where anabolic outcomes were the primary focus [49-51]. In contrast, the BCAA dose in the OptHealth study was specifically selected to modify the daily dietary AA ratio rather than to maximize muscle anabolism. The primary justification for this dosage was to increase the daily BCAA:tryptophan ratio to that which induced hyperphagia in mice [15]. This approach reflects a direct translation of preclinical findings, demonstrating that changes in AA balance, rather than total BCAA intake, influence appetite-related outcomes.

From a clinical perspective, declining appetite with advancing age is associated with reduced nutrient intake and resulting risks of malnutrition, frailty, and other age-related conditions, thereby impacting overall health and quality of life. Given that there is still a lack of pharmacological treatment for appetite decline in the aging population, this study may provide a novel approach based on nutritional intervention by translating findings from our previous animal research into humans, potentially addressing both appetite regulation and broader health challenges associated with aging.

### Strength and Limitations

To our knowledge, the OptHealth study is the first clinical trial to investigate the relationship between BCAAs and appetite regulation in humans. The controlled randomized trial design is considered the gold standard for generating clinical evidence, as it minimizes bias and allows for robust assessment of causal relationships between dietary interventions and measured outcomes. The dietary intervention represents a low-risk, nonpharmaceutical approach, increasing its translational value for the development of practical nutritional strategies that may be more acceptable and sustainable for older adults. Another key strength of the study is the inclusion of multiple intervention arms. Apart from the control and a BCAA-only supplementation group, the trial incorporated BCAA combined with tryptophan and methionine. This design allows for direct testing of hypotheses generated from animal models and strengthens the capacity to interpret observed effects beyond BCAAs alone.

However, there are several limitations that should be considered. First, the 4-week intervention duration was selected to balance feasibility and participant compliance; however, this relatively short time frame may not capture the long-term metabolic adaptations or health outcomes associated with chronic BCAA supplementation. Second, the study set out specifically to recruit generally healthy older adults, which may limit the generalizability of the findings to populations with pre-existing chronic diseases, sarcopenia, or significant malnutrition. Future studies will be required to determine whether similar effects are observed in more diverse or clinical cohorts. Finally, due to the nature of dietary intervention and supplied supplementation, blinding was not feasible, resulting in an open-label study design. This lack of blinding may introduce performance or expectation bias, especially for self-reported outcomes. To mitigate potential bias, the study uses several rigorous controls: participants were randomly allocated to intervention groups, all groups received a standardized study diet, and study procedures and participant contact are standardized across groups. In addition, the inclusion of objective metabolic biomarkers such as circulating FGF-21 provides complementary outcome measures that are not susceptible to reporting bias.

### Dissemination Plan

The findings from this study will be disseminated through peer-reviewed publications in journals focused on nutrition, aging, and clinical research to reach academic and clinical audiences. Results will also be presented at national and international conferences relevant to nutrition, obesity, and aging. In addition, study findings may be shared with key stakeholders, health professionals, and the general public through seminars, workshops, or public engagement activities to support knowledge translation and inform evidence-based dietary practices.

### Conclusions

In the context of global population aging, obesity-related chronic conditions require increasing attention. By translating evidence from animal models into human research, this trial aims to address the important gap in understanding the role of dietary AA profiles in appetite control, specifically whether the hyperphagic effects observed with high-BCAA diets in mice are also evident in humans. This study may provide a novel source of nutritional interventions, potentially addressing both appetite regulation and broader health challenges associated with aging.

## Supplementary material

10.2196/82436 Multimedia Appendix 1 Detailed nutrition profile of provided study foods.

10.2196/82436 Checklist 1 SPIRIT checklist.

## Abbreviations

7dWFR: 7-day weighed food record
AA: amino acid
BCAA: branched-chain amino acid
CPC: Charles Perkins Centre
FGF-21: fibroblast growth factor 21
IGF-1: insulin-like growth factor 1
OptHealth: Optimizing Diet for Better Health
RCT: randomized clinical trial
REDCap: Research Electronic Data Capture
RPAH: Royal Prince Alfred Hospital
SPIRIT: Standard Protocol Items: Recommendations for Interventional Trials
