# Supplementary material for: Effect of Branched-Chain Amino Acid Supplementation Alone or Combined With Tryptophan or Methionine on Appetite Control and Related Health Outcomes in Older Adults: Protocol for a Randomized Controlled Trial
Source: JMIR Res Protoc. 2026 May 21;15:e82436. doi: 10.2196/82436 (PMC13193670; doi:10.2196/82436)
Supplement: Multimedia Appendix 1 [file resprot-v15-e82436-s001.docx]

| Nutrients | |
| --- | --- |
| Energy, kJ | 6708.6 |
| Energy, Cal | 1602.6 |
| Protein, g | 85.2 |
| Total fat, g | 45.4 |
| Saturated fat, g | 14.5 |
| Carbohydrate, g | 197.5 |
| Sugar, g | 70.7 |
| Starch, g | 122.3 |
| Dietary fiber, g | 31.0 |
| Sodium, mg | 1628.8 |
| Energy from protein, % | 21.6 |
| Energy from fat, % | 25.0 |
| Energy from saturated fat, % | 8.0 |
| Energy from carbohydrate, % | 47.9 |
| Energy from fiber, % | 3.7 |

Detailed nutrition profile with provided study foods.
